# Cyclopædia of the Practice of Medicine

**Published:** 1876-09

**Authors:** 


Cyclopœdia of the Practice of Medicine. Edited by H. Von Ziemssen, Professor of Clinical Medicine in Munich, Bavaria. Vol. xi. Diseases of the Peripheral Cerebro-Spinal Nerves. By Prof. Wilhelm Heinrich Erb, of Heidelberg, Baden. Translated by Henry Parver, of London, England. Albert H. Buck, M.D., New York, Editor American Edition. William Wood & Co., 27 Great Jones street, New York. 1876.

The eleventh volume of this great work is at hand, and is, in some particulars, an improvement upon the preceding ten volumes. The entire volume of 600 pages is devoted to the diseases of the peripheral cerebro-spinal nerves. The great advance in neuropathology within the past ten or fifteen years makes such a work as this a desideratum to the profession. As the improvement in this department of medicine has been the result of an accumulation of accurate descriptions of clinical phenomena and the appropriate remedial measures, which have usually been presented to the profession through the medical press in the form of monographs, the true status of our knowledge upon this class of diseases has not been well defined. In this volume we have the chaff separated from the grain, in the form of an accurate record of the present state of the science upon the pathology, etiology and treatment of the diseases of the peripheral cerebro-spinal nerves.

The first one hundred and ninety pages are devoted to neuralgia, embracing neuralgia in general, neuralgia of the individual nerves, neuralgia of the fifth, cerebro-occipital neuralgia, neuralgia of the brachial plexus, of the dorsal nerves, of the plexus lumbalis, of the plexus sacralis, and plexus coccygeus. The next forty pages is devoted to anæsthesia. Next comes the discussion of the neuroses of the nerves of special sense, embracing the neuroses of the nerves of taste, and of the olfactory nerve. The next three hundred and thirteen pages are devoted to the neuroses of the nerves of motion, in which is discussed spasms, or convulsions in general, and paralysis, embracing the particular forms of spasm; first, as to its character as tonic, clonic, cramps, etc.; and secondly, spasms of the distribution of particular nerves or sets of nerves or plexuses, as spasms of the distribution of the facial nerve, spasm of the respiratory nerves, etc. The different forms of paralysis, the result of a neurosis of the nerves of motion, both as to character and location, receive a due share of attention. The last thirty pages are devoted to the anatomical diseases of the peripheral nerves, or as hyperæmia, inflammation, atrophy, and hypertrophy of the nerves, including neoplastic formations in the nerves.

As we have before said, we regard the Cyclopædia as the greatest work on the practice of medicine in the English language. The profession will ever be under obligation to Messrs. Wood & Co. for this most valuable contribution.

